# Instrumented insoles for assessment of gait in patients with vestibular schwannoma

**DOI:** 10.1017/wtc.2023.11

**Published:** 2023-05-10

**Authors:** Stephen Leong, Bing M. Teh, Ton Duong, Diane Hu, Alexander Chui, Jocelyn S. Chen, Michael B. Sisti, Tony J.C. Wang, Damiano Zanotto, Anil K. Lalwani

**Affiliations:** 1 Vagelos College of Physicians & Surgeons, Columbia University Irving Medical Center, New York, NY, USA; 2 Department of Otolaryngology—Head & Neck Surgery, NewYork-Presbyterian/Columbia University Irving Medical Center, New York, NY, USA; 3 Department of Otolaryngology—Head & Neck Surgery, Monash Health; Faculty of Medicine, Nursing and Health Sciences, Monash University, Clayton, VC, Australia; 4 Department of Mechanical Engineering, Stevens Institute of Technology, Hoboken, NJ, USA; 5 Department of Biological Sciences, Columbia University, New York, NY, USA; 6 Department of Biomedical Engineering, Columbia University, New York, NY, USA; 7 Department of Neurological Surgery, NewYork-Presbyterian/Columbia University Irving Medical Center, New York, NY, USA; 8 Department of Radiation Oncology, NewYork-Presbyterian/Columbia University Irving Medical Center, New York, NY, USA; 9 Department of Mechanical Engineering, Columbia University, New York, NY, USA

**Keywords:** Sensors, soft wearable robotics, vestibular schwannoma, vestibular dysfunction

## Abstract

**Background:**

Imbalance and gait disturbances are common in patients with vestibular schwannoma (VS) and can result in significant morbidity. Current methods for quantitative gait analysis are cumbersome and difficult to implement. Here, we use custom-engineered instrumented insoles to evaluate the gait of patients diagnosed with VS.

**Methods:**

Twenty patients with VS were recruited from otology, neurosurgery, and radiation oncology clinics at a tertiary referral center. Functional gait assessment (FGA), 2-minute walk test (2MWT), and uneven surface walk test (USWT) were performed. Custom-engineered instrumented insoles, equipped with an 8-cell force sensitive resistor (FSR) and a 9-degree-of-freedom inertial measurement unit (IMU), were used to collect stride-by-stride spatiotemporal gait parameters, from which mean values and coefficients of variation (CV) were determined for each patient.

**Results:**

FGA scores were significantly correlated with gait metrics obtained from the 2MWT and USWT, including stride length, stride velocity, normalized stride length, normalized stride velocity, stride length CV, and stride velocity CV. Tumor diameter was negatively associated with stride time and swing time on the 2MWT; no such association existed between tumor diameter and FGA or DHI.

**Conclusions:**

Instrumented insoles may unveil associations between VS tumor size and gait dysfunction that cannot be captured by standardized clinical assessments and self-reported questionnaires.

## 1. Introduction

Vestibular schwannomas (VS) are benign tumors of the vestibulocochlear nerve that may present with unilateral hearing loss, tinnitus, episodic vertigo, and imbalance (Kentala and Pyykkö, 2001). Additionally, patients with VS often have some level of gait disturbance, although most patients achieve a level of vestibular compensation such that their disequilibrium is tolerable on a daily basis (Ishikawa et al., 2001, 2004; Kentala and Pyykkö, 2001; Angunsri et al., 2011; Wang et al., 2011; Yin et al., 2011; Nam et al., 2018). Nonetheless, gait disturbance is a significant risk factor for falls, especially in elderly patients, and the link between gait disturbance and fall risk has been identified for multiple domains of gait (Hausdorff et al., 2001; Cesari et al., 2005; Springer et al., 2006; Abellan van Kan et al., 2009; Nordin et al., 2010; Oh-Park et al., 2011; Studenski et al., 2011; Pamoukdjian et al., 2015). Specifically, gait speed, stride length, and gait variability have been shown to be correlated with frailty and overall survival in elderly patients (Hausdorff et al., 2001; Springer et al., 2006; Abellan van Kan et al., 2009; Nordin et al., 2010; Studenski et al., 2011; Pamoukdjian et al., 2015). In patients with VS, increased gait variability and greater foot pressure on the side of the lesion have been demonstrated, particularly with visual deprivation (Ishikawa et al., 2001, 2004; Angunsri et al., 2011; Wang et al., 2011; Yin et al., 2011).

Previous studies have utilized clinical assessments and standardized surveys to assess gait in patients with VS or vestibular disorders; in particular, the Functional Gait Assessment (FGA) and Dizziness Handicap Index (DHI) have been used as strong predictors of quality-of-life in patients with VS receiving surgical treatment (Said et al., 2021; Zobeiri et al., 2021). The DHI is a 25-item self-reported questionnaire that quantifies patients’ dizziness disability in three domains: functional, emotional, and physical (Jacobson and Newman, 1990). In contrast, the FGA is a 10-part series of ambulation tasks, where patients walk with eyes closed, over an obstacle, over a set of stairs, etc. (Wrisley et al., 2004). Though both assessments have had utility in the VS population, the DHI is more commonly used due to its ease of administration (Mutlu and Serbetcioglu, 2013; Zobeiri et al., 2021). The FGA has been validated against other measures of dizziness, balance, and fall risk (Wrisley and Kumar, 2010) and provides more comprehensive data on patients’ walking capacity (Marchetti et al., 2014).

Recently, the assessment of gait in patients with VS has involved a number of gait parameters, including the trajectory of the center of force (TCOF), foot pressure, stance time, swing time, and double support time, along with the coefficient of variation (CV) associated with each of these metrics (Ishikawa et al., 2004; Angunsri et al., 2011; Yin et al., 2011). Notably, studies on these parameters have demonstrated that patients with VS do not have significant gait phase changes, but do have a higher CV for stance and swing when walking with eyes closed (Wang et al., 2011; Yin et al., 2011). Additionally, patients with VS exhibit a gait shift to the side of their lesion when walking with their eyes closed, demonstrating the role of visual feedback in producing compensation (Ishikawa et al., 2004).

Quantitative gait analysis for patients with VS may allow for early detection of imbalance and disequilibrium and early initiation of physical and vestibular therapy. However, traditional equipment for gait analysis is expensive and/or cumbersome and cannot easily be applied to the clinical setting. Examples of devices used in previous studies include non-reusable tactile sensors attached to patients’ feet with adhesive tape (Ishikawa et al., 2001; Angunsri et al., 2011), instrumented walkways (Ohara et al., 2021), and optical motion analysis systems (Anson et al., 2019), none of which can easily be introduced into office spaces. Additionally, these systems offer only a limited working distance (8–10 m) over which patients can navigate, when in fact dozens to hundreds of strides may be required to reliably evaluate gait variability (Hollman et al., 2010; Lord et al., 2011).

Our team has previously developed various iterations of instrumented footwear for the assessment of gait and balance, all of which consist of highly portable sets of inertial sensors embedded in footwear (Zanotto et al., 2014; Minto et al., 2016; Zhang et al., 2022). We have used this footwear to explore associations between gait disturbances and DHI scores (Zanotto et al., 2017), and to demonstrate that hearing loss is highly correlated with gait variability, which highlights the importance of auditory feedback for walking and balance (Szeto et al., 2021). Despite the usability of our technology, patient comfort may have been a limiting factor—our technology thus far has required patients to wear instrumented sandals in place of their own footwear to complete gait analysis, which may have affected their natural walking patterns. In this study, we use new, minimally obtrusive instrumented insoles that may be fitted into the patients’ own footwear, which increases both portability and comfort during walking tasks (Duong et al., 2022; Zhang et al., 2017, 2020). Specifically, we use instrumented insoles to evaluate gait in patients with VS; we perform the FGA as a standardized assessment, then collect insole data during a 2-minute walk test (2MWT) and an uneven surface walk test (USWT). The 2MWT is a validated test commonly used to evaluate functional endurance in adults (Bohannon et al., 2015); variations of the USWT have frequently been used to assess gait dysfunction in the elderly (Bogen et al., 2019; Osoba et al., 2019). Used in combination with the instrumented insoles, the 2MWT allows a large number of steady-state footsteps to be recorded; the USWT test may emphasize gait and balance dysfunction in patients with VS due to the challenging walking surface.

## 2. Materials and methods

### 2.1. Patient recruitment

Patients presenting to otology, neurosurgery, and radiation oncology clinics in a tertiary care setting were evaluated via chart review for an active diagnosis of VS. Patients with a significant neurologic disorder, diagnosis of neurofibromatosis II, and age > 80 years were excluded from the study. Patients fulfilling both inclusion and exclusion criteria were contacted via phone and asked for voluntary participation in the study; a confirmation email was sent to all patients agreeing to participate. All study procedures and details were approved by the Institutional Review Board (IRB) of Columbia University.

### 2.2. Instrumented insoles

**Figure 1.**
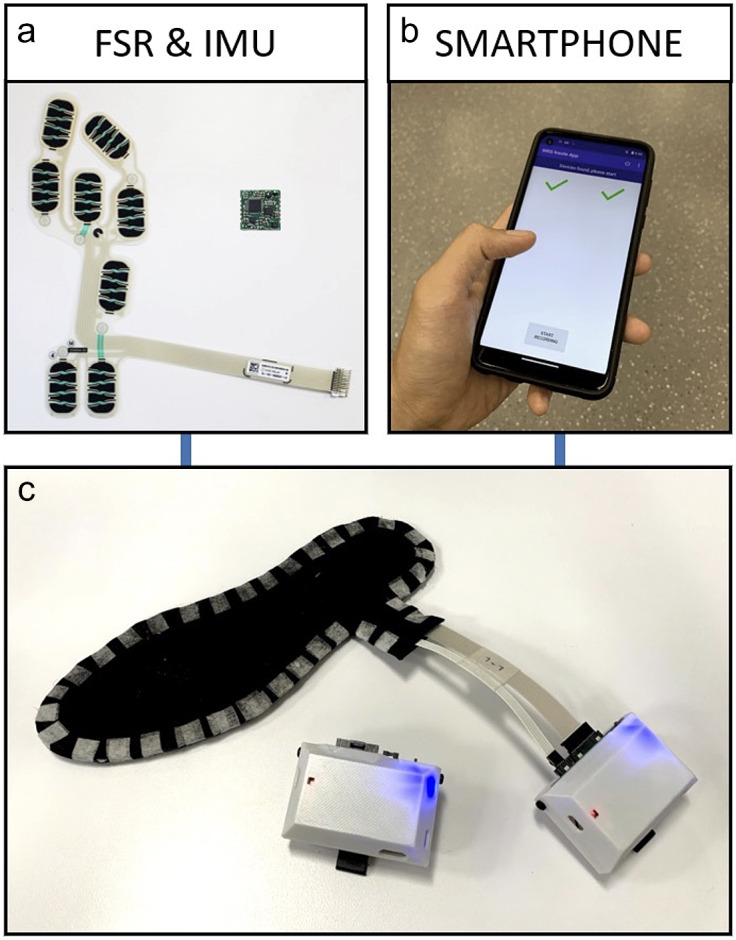
Components of the instrumented insole system, including (a) an 8-cell force sensitive resistor (FSR) and 9-degree-of-freedom inertial measurement unit (IMU), (b) a smartphone control application, and (c) the fully assembled insole and logic unit.

The instrumented insole system was developed to capture spatiotemporal gait parameters in real-life environments (Duong et al., 2022). The system consists of a pair of instrumented insoles, a pair of logic units clipped to the posterolateral side of the patient’s footwear, and an Android smartphone (Figure 1). Each insole consists of an 8-cell force-sensitive resistor (FSR) and a 9-degree-of-freedom inertial measurement unit (IMU). The logic unit consists of a Linux-based single-board computer with integrated Wi-Fi connectivity and on-board data storage. Each logic unit is powered by a 3.7 V 2000mAh Li-Po battery through a 5 V voltage booster with built-in charger circuit. Data from the instrumented insoles are acquired by the logic units at 333 Hz. The system is controlled by the Android smartphone through a custom application. In this study, four insole sizes were used to cover most of the common US shoe sizes (US W5.5 to M11). The overall weight of an instrumented insole and a logic unit is less than 130 grams. The hardware and software require approximately 5 minutes to equip onto patients and initiate data collection. This technology has been validated against gold-standard gait analysis systems in young healthy individuals (Zhang et al., 2020), older adults (Zhang et al., 2022), and patients with neuromuscular (Duong et al., 2021) or neurodevelopmental (Duong et al., 2020) disorders.

### 2.3. Gait and balance assessments

Patients participating in the study completed an online Qualtrics survey (Qualtrics XM, Seattle, Washington) consisting of demographic questions (age, sex, race, height, medical history) and the standard 25-question DHI prior to in-person testing. For all gait assessments, patients wore instrumented insoles for the duration of testing. Patients completed three standardized gait assessments, including the 10-part FGA (Wrisley et al., 2004), the 2-minute walk test (Bohannon et al., 2015), and an 80-meter (10-meter walkway, 8 laps) uneven surface walk test performed on two 8 feet x 4 feet x 2 inch soft exercise mats (Bogen et al., 2019). Gait parameters obtained from insoles included stride length, stride time, stride velocity, stance percent, and swing percent. Gait parameters were obtained from the USWT and 2MWT due to the extended duration of walking required for both tests. Normalization for stride length and stride velocity were calculated as previously described (Hof, 1996). Following testing, the mean and CV for each metric was calculated for all patients.

### 2.4. Statistical analysis

All statistical analyses were performed in R (R Core Team, Vienna, Austria) and Prism (GraphPad, San Diego, California). Patient data were approximately normal in distribution. Pearson correlation analyses were performed to compare scores on FGA and DHI versus insole-derived gait parameters. Linear regression was used to analyze the relationship between tumor size and DHI, FGA, and gait parameters. Paired t-tests were performed to compare gait parameters obtained during the 2MWT and the USWT.

## 3. Results

In total, 20 patients with a diagnosis of VS completed gait testing with instrumented insoles (Table 1). Of these patients, 20 completed USWT, 16 completed 2MWT, and 16 completed the online survey, including the DHI. The mean age among participants was 63.0 ± 10.1; participants were predominantly female (60.0%). Of the patients completing the online survey, 75.0% reported hearing loss, 63.5% reported tinnitus, and 50.0% reported dizziness (Table 1). The mean DHI score among participants was 14.0 ± 16.1; the mean FGA score was 25.1 ± 3.9; and the mean tumor diameter was 13.5 mm ± 6.8 mm (Table 1).

**Table 1.** Demographics and characteristics of the study population

	Number (%)
*Total patients completing gait testing*	20 (100%)
Age (Mean ± SD)	63.0 ± 10.1
Female	12 (60.0%)
Male	8 (40.0%)
*Total patients completing email survey*	16 (80.0%)
Reporting hearing loss	12 (75.0%)
Reporting tinnitus	10 (62.5%)
Reporting dizziness	8 (50.0%)
Reporting fall(s)	3 (18.8%)
Past ophthalmologic history	5 (31.3%)
Past neurologic history	3 (18.8%)
Past other balance disorder	2 (12.5%)
Average DHI (Mean ± SD)	14.0 ± 16.1
Average FGA (Mean ± SD)	25.1 ± 3.9
Average tumor diameter (mm) (Mean ± SD)	13.5 ± 6.8

*Note.* In total, 20 patients completed some amount of gait testing; of these 20 patients, 16 completed the online survey. Symptomatology and past medical history are based on the 16-patient group; other demographics and scores are based on the 20-patient group, with the exception of the DHI (based on the 16-patient group).

**Figure 2.**
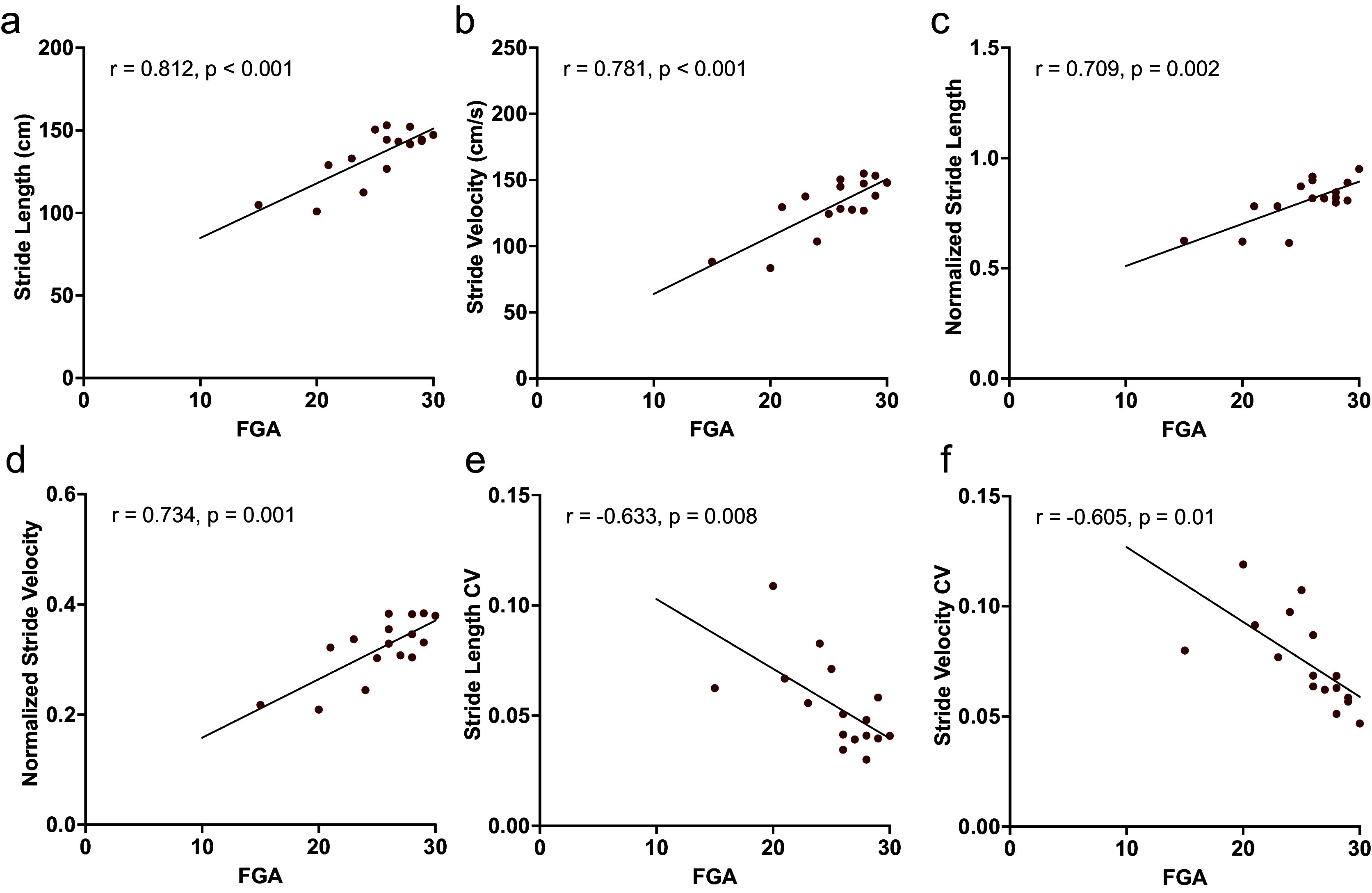
Pearson correlation analyses for FGA versus selected gait parameters obtained from the 2MWT. All selected parameters are significantly correlated with FGA, and include stride length (a), stride velocity (b), normalized stride length (c), normalized stride velocity (d), stride length CV (e), and stride velocity CV (f). Correlation coefficients and corresponding p-values are displayed.

DHI score was significantly correlated with the following 2MWT metrics: swing time (*r =* −0.592, *p =* 0.03) and stride time CV (*r =* 0.719, *p =* 0.006) (Supplementary Tables S1 and S2). FGA score was significantly correlated with the following 2MWT and USWT metrics: stride length (2MWT: *r =* 0.81, *p =* 0.0001; USWT: *r =* 0.78, *p =* 0.00004), stride velocity (2MWT: *r =* 0.78, *p =* 0.0003; USWT: *r =* 0.72, *p =* 0.0004), normalized stride length (2MWT: *r =* 0.71, *p =* 0.002; USWT: *r =* 0.73, *p =* 0.0003), normalized stride velocity (2MWT: *r =* 0.73, *p =* 0.001; USWT: *r =* 0.67, *p =* 0.001), stride length CV (2MWT: *r =* −0.63, *p =* 0.008; USWT: *r =* −0.57, *p =* 0.009), and stride velocity CV (2MWT: *r =* −0.61, *p =* 0.01, USWT: *r =* −0.56, *p =* 0.01) (Figure 2, Supplementary Tables S1 and S2).

**Figure 3.**
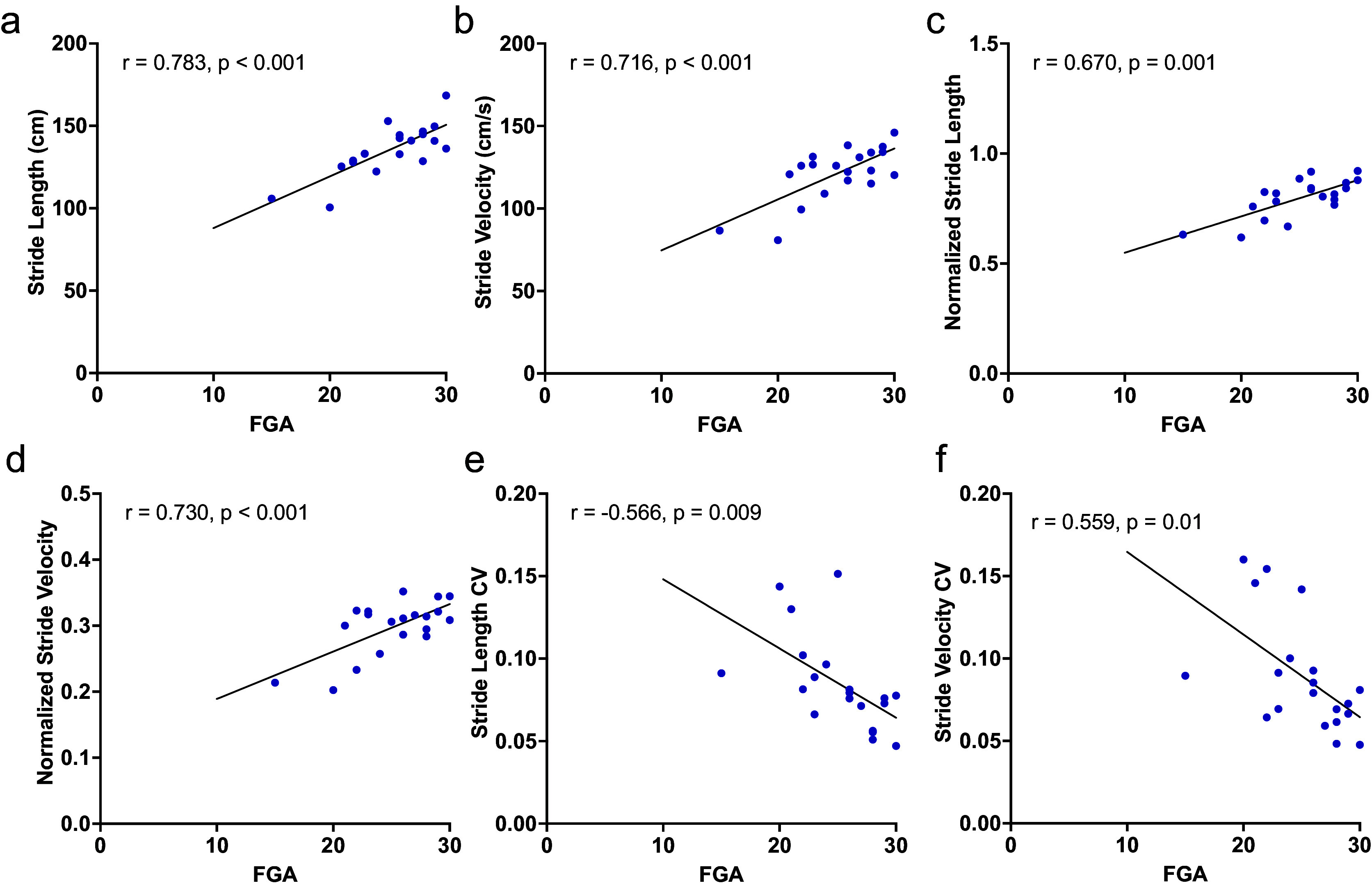
Pearson correlation analyses for FGA versus selected gait parameters obtained from the USWT. All selected parameters are significantly correlated with FGA, and include stride length (a), stride velocity (b), normalized stride length (c), normalized stride velocity (d), stride length CV (e), and stride velocity CV (f). Correlation coefficients and corresponding p-values are displayed.

**Figure 4.**
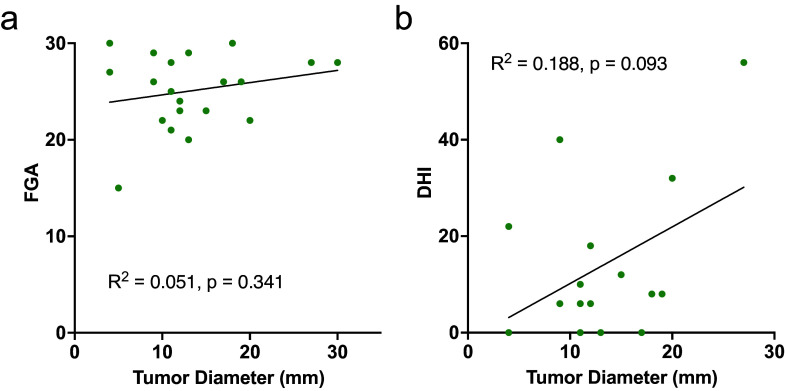
Linear regression analyses for tumor diameter versus FGA (a) and DHI (b) scores. Neither FGA nor DHI are significantly associated with tumor size. *R* ^2^ values and corresponding *p*-values are displayed.

**Figure 5.**
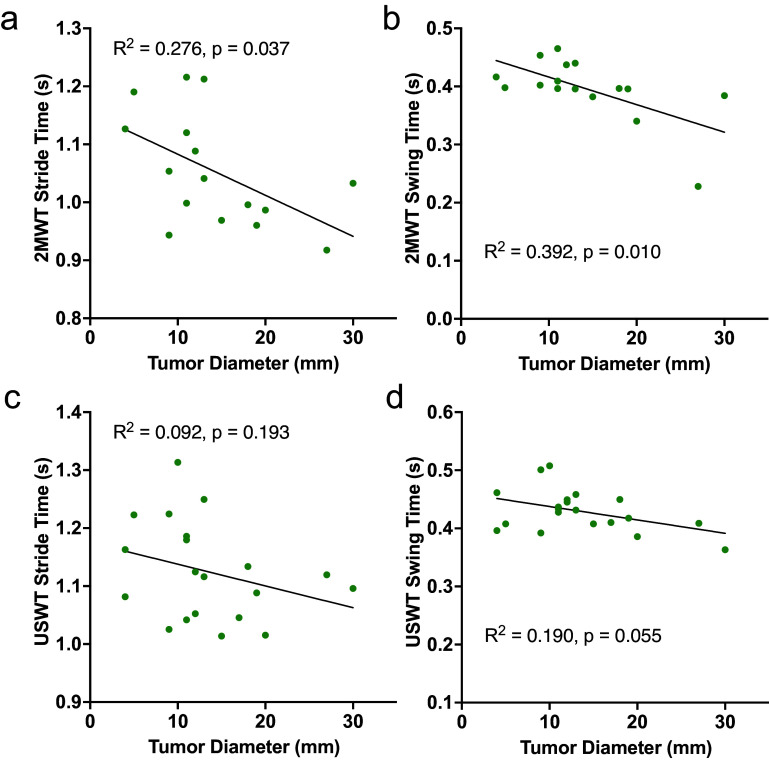
Linear regression analyses for tumor diameter versus 2MWT metrics: stride time (a) and swing time (b), and USWT metrics: stride time (c) and swing time (d). Neither FGA nor DHI are significantly associated with tumor size. *R* ^2^ values and corresponding *p*-values are displayed.

No linear relationship existed between tumor diameter and FGA score (*R* ^2^ = 0.05, *p =* 0.3) or DHI score (*R* ^2^ = 0.19, *p =* 0.09) (Figure 4). Linear relationships existed between tumor diameter and the following 2MWT metrics: stride time (*R* ^2^ = 0.28, *p =* 0.04) and swing time (*R* ^2^ = 0.39, *p =* 0.01) (Figure 5; Supplementary Table S3). No linear relationships existed between tumor diameter and USWT metrics, although the regression analysis for swing time approached significance (*R* ^2^ = 0.190, *p =* 0.055) (Figure 5; Supplementary Table S3).

## 4. Discussion

In this study, we used instrumented insoles to assess gait in patients with an active diagnosis of VS. The insoles are highly portable and can be used with the patient’s own footwear to maximize comfort. Additionally, the system is relatively straightforward to initiate, requiring only 5 minutes to equip onto patients for data collection, including software start-up time. Thus, instrumented insoles may easily be integrated into the routine management of patients with VS, in the clinical setting and potentially in the home setting as well.

In our patient population, the average FGA score was 25.1 ± 3.9, comparable to the national average for the age group (Walker et al., 2007). The average DHI score was 14.0 ± 16.1, corresponding to mild levels of dizziness (Formeister et al., 2020). Most patients reported hearing loss and/or tinnitus. These results are consistent with the most common presentation of VS, where hearing symptoms predominate and vestibular symptoms are relatively mild (Kentala and Pyykkö, 2001). We found that FGA scores were strongly positively correlated with a number of gait parameters, namely, stride length, stride velocity, normalized stride length, and normalized stride velocity, for both 2MWT and USWT. These results are consistent with the literature, as patients with vestibular gait dysfunction have worse FGA performance, with worse balance, dizziness, and fall risk (Marchetti et al., 2014), and in general take shorter strides (Yamamoto et al., 2002; Agrawal et al., 2013; Chae et al., 2021). Thus, we demonstrate that our instrumented insoles can reliably be used to detect walking dysfunction. The moderate negative correlation between FGA and the coefficient of variability for stride length and stride velocity adds additional evidence to this notion, since increased variability in gait is expected in patients with vestibular gait dysfunction (Hausdorff et al., 2001; Lord et al., 2011; Szeto et al., 2021). Interestingly, we did not find comparable correlations between the DHI and insole-derived gait parameters, which may be due to the highly subjective nature of the DHI. The subjectivity of the DHI is a particular concern for patients with VS, who may not necessarily characterize their vestibular dysfunction as dizziness (Kentala and Pyykkö, 2001).

Among our patients, the average VS tumor diameter was 13.5 mm ± 6.8 mm, suggesting that most patients presented early in the course of their disease (Sughrue et al., 2010). We found no linear associations between tumor diameter and FGA or DHI scores, likely due to ceiling effects for both assessments. Among gait parameters, we found that tumor diameter was negatively associated with two 2MWT gait parameters, stride time and swing time. In other words, patients with larger tumors had shorter swing periods, producing shorter stride times. This association was weaker during the USWT, though in general patients took slower steps on the uneven surface. These results indicate that tumor size may be related to gait dysfunction, as is consistent with the literature (Wagner et al., 2011; Yin et al., 2011), though the exact type of dysfunction is unclear. Thus, instrumented insoles may be used to characterize subtle changes in gait associated with tumor growth that the FGA and DHI cannot detect.

The major strength of our study was the use of innovative technology in a patient population with a specific medical diagnosis. By doing so, we accomplished two goals: we provided further evidence for the utility of our technology, and we drew conclusions about gait dysfunction in patients with VS. Major limitations in our study included non-responses on the online survey and inability to complete gait testing for some patients. Additionally, our study lacked an age-matched control group for comparison, which would have allowed for more robust conclusions to be drawn from the data. Given the usability and portability of our instrumented insoles, assembling a reference group will likely be straightforward, especially if devices may be distributed to patients for testing in the home setting.

## 5. Conclusion

In this study, we used instrumented insoles to assess gait in patients with an active diagnosis of VS. We found significant correlations between insole-derived gait parameters and FGA scores, indicating that insole data was consistent with validated tests routinely used in the clinical setting. Though tumor size was not a reliable predictor of FGA or DHI scores, tumor size was negatively associated with stride time and swing time on the 2MWT test. Thus, instrumented insoles may unveil associations between tumor growth and gait dysfunction that cannot be captured by standardized clinical assessments and self-reported questionnaires.

## Data Availability

Full datasets have been made available in the supplementary materials. Protocols may be requested through the corresponding author.

## Supplementary material

For supplementary material accompanying this paper visit https://doi.org/10.1017/wtc.2023.11. click here to view supplementary material
