# Supplementary material for: Instrumented insoles for assessment of gait in patients with vestibular schwannoma
Source: Wearable Technol. 2023 May 10;4:e14. doi: 10.1017/wtc.2023.11 (PMC10936291; doi:10.1017/wtc.2023.11)
Supplement: Supplementary file 1 [file wtcsup.zip › S2631717623000117sup001.docx]

Supplementary Table 1. Pearson correlation analyses for 2MWT gait parameters vs. FGA and DHI. Correlation coefficients are displayed in the second column, and associated p-values are displayed in the third.

| 2MWT gait parameter | FGA | p-value |
| --- | --- | --- |
| Stride time (s) | -0.466 | 0.0688 |
| Stride length (cm) | **0.812** | **0.000133** |
| Stride velocity (cm/s) | **0.781** | **0.000356** |
| Normalized stride length | **0.709** | **0.00212** |
| Normalized stride velocity | **0.734** | **0.00121** |
| Swing time (s) | -0.154 | 0.570 |
| Swing percent | 0.144 | 0.594 |
| Stance time (s) | -0.442 | 0.0865 |
| Stance percent | -0.144 | 0.594 |
| Stride time CV | 0.0689 | 0.800 |
| Stride length CV | **-0.633** | **0.00843** |
| Stride velocity CV | **-0.605** | **0.0131** |
| Swing time CV | -0.0193 | 0.944 |
| Swing percent CV | 0.102 | 0.708 |
| Stance time CV | 0.00202 | 0.994 |
| Stance percent CV | 0.125 | 0.644 |

| 2MWT gait parameter | DHI | p-value |
| --- | --- | --- |
| Stride time (s) | -0.342 | 0.253 |
| Stride length (cm) | -0.0346 | 0.911 |
| Stride velocity (cm/s) | 0.242 | 0.426 |
| Normalized stride length | 0.0586 | 0.849 |
| Normalized stride velocity | 0.243 | 0.424 |
| Swing time (s) | **-0.592** | **0.0332** |
| Swing percent | -0.509 | 0.0756 |
| Stance time (s) | 0.0446 | 0.885 |
| Stance percent | 0.509 | 0.0756 |
| Stride time CV | **0.719** | **0.00558** |
| Stride length CV | -0.143 | 0.642 |
| Stride velocity CV | -0.0693 | 0.822 |
| Swing time CV | 0.428 | 0.145 |
| Swing percent CV | 0.485 | 0.0931 |
| Stance time CV | 0.293 | 0.331 |
| Stance percent CV | 0.355 | 0.235 |
