# Supplementary material for: Instrumented insoles for assessment of gait in patients with vestibular schwannoma
Source: Wearable Technol. 2023 May 10;4:e14. doi: 10.1017/wtc.2023.11 (PMC10936291; doi:10.1017/wtc.2023.11)
Supplement: Supplementary file 1 [file wtcsup.zip › S2631717623000117sup002.docx]

Supplementary Table 2. Pearson correlation analyses for USWT gait parameters vs. FGA and DHI. Correlation coefficients are displayed in the second column, and associated p-values are displayed in the third.

| USWT gait parameter | FGA | p-value |
| --- | --- | --- |
| Stride time (s) | -0.222 | 0.346 |
| Stride length (cm) | **0.784** | **0.0000438** |
| Stride velocity (cm/s) | **0.716** | **0.000388** |
| Normalized stride length | **0.730** | **0.000258** |
| Normalized stride velocity | **0.670** | **0.00124** |
| Swing time (s) | -0.0712 | 0.766 |
| Swing percent | 0.0742 | 0.756 |
| Stance time (s) | -0.202 | 0.392 |
| Stance percent | -0.0742 | 0.756 |
| Stride time CV | -0.261 | 0.267 |
| Stride length CV | **-0.566** | **0.00935** |
| Stride velocity CV | **-0.559** | **0.0105** |
| Swing time CV | -0.183 | 0.439 |
| Swing percent CV | -0.128 | 0.591 |
| Stance time CV | -0.321 | 0.167 |
| Stance percent CV | -0.140 | 0.557 |

| USWT gait parameter | DHI | p-value |
| --- | --- | --- |
| Stride time (s) | 0.248 | 0.355 |
| Stride length (cm) | -0.198 | 0.462 |
| Stride velocity (cm/s) | -0.400 | 0.125 |
| Normalized stride length | -0.221 | 0.410 |
| Normalized stride velocity | -0.386 | 0.140 |
| Swing time (s) | 0.188 | 0.486 |
| Swing percent | -0.00307 | 0.991 |
| Stance time (s) | 0.134 | 0.620 |
| Stance percent | 0.00307 | 0.991 |
| Stride time CV | 0.254 | 0.343 |
| Stride length CV | -0.198 | 0.462 |
| Stride velocity CV | -0.111 | 0.682 |
| Swing time CV | -0.00706 | 0.979 |
| Swing percent CV | -0.0379 | 0.889 |
| Stance time CV | -0.145 | 0.593 |
| Stance percent CV | -0.0443 | 0.871 |
