# Supplementary material for: Instrumented insoles for assessment of gait in patients with vestibular schwannoma
Source: Wearable Technol. 2023 May 10;4:e14. doi: 10.1017/wtc.2023.11 (PMC10936291; doi:10.1017/wtc.2023.11)
Supplement: Supplementary file 1 [file wtcsup.zip › S2631717623000117sup003.docx]

Supplementary Table 3. Simple linear regression analyses for USWT and 2MWT gait parameters vs. tumor diameter (mm). R^2^ values are displayed in the second column, and associated p-values are displayed in the third.

| 2MWT gait parameter | R^2^ | p-value |
| --- | --- | --- |
| Stride time (s) | **0.276** | **0.0365** |
| Stride length (cm) | 0.0722 | 0.314 |
| Stride velocity (cm/s) | 0.219 | 0.0675 |
| Normalized stride length | 0.0749 | 0.305 |
| Normalized stride velocity | 0.208 | 0.0758 |
| Swing time (s) | **0.392** | **0.00950** |
| Swing percent | 0.136 | 0.159 |
| Stance time (s) | 0.0443 | 0.434 |
| Stance percent | 0.127 | 0.176 |
| Stride time CV | 0.0304 | 0.518 |
| Stride length CV | 0.0976 | 0.239 |
| Stride velocity CV | 0.0874 | 0.266 |
| Swing time CV | 0.0419 | 0.447 |
| Swing percent CV | 0.0163 | 0.638 |
| Stance time CV | 0.0318 | 0.509 |
| Stance percent CV | 0.00750 | 0.750 |

| USWT gait parameter | R^2^ | p-value |
| --- | --- | --- |
| Stride time (s) | 0.0924 | 0.193 |
| Stride length (cm) | 0.00233 | 0.840 |
| Stride velocity (cm/s) | 0.0113 | 0.656 |
| Normalized stride length | 0.00458 | 0.777 |
| Normalized stride velocity | 0.0222 | 0.531 |
| Swing time (s) | 0.190 | 0.0547 |
| Swing percent | 0.0714 | 0.255 |
| Stance time (s) | 0.00857 | 0.698 |
| Stance percent | 0.0714 | 0.255 |
| Stride time CV | 0.0711 | 0.256 |
| Stride length CV | 0.0465 | 0.361 |
| Stride velocity CV | 0.0449 | 0.370 |
| Swing time CV | 0.0191 | 0.561 |
| Swing percent CV | 0.000186 | 0.955 |
| Stance time CV | 0.0656 | 0.276 |
| Stance percent CV | 0.00935 | 0.685 |
